# A Deep Learning Framework for Automatic Sleep Apnea Classification Based on Empirical Mode Decomposition Derived from Single-Lead Electrocardiogram

**DOI:** 10.3390/life12101509

**Published:** 2022-09-27

**Authors:** Febryan Setiawan, Che-Wei Lin

**Affiliations:** 1 Department of Biomedical Engineering, College of Engineering, National Cheng Kung University, Tainan 701, Taiwan; 2 Institute of Gerontology, College of Medicine, National Cheng Kung University, Tainan 701, Taiwan; 3 Medical Device Innovation Center, National Cheng Kung University, Tainan 701, Taiwan; 4 Institute of Medical Informatics, College of Electrical Engineering and Computer Science, National Cheng Kung University, Tainan 701, Taiwan

**Keywords:** sleep apnea detection, single-lead electrocardiogram, empirical mode decomposition, deep learning, imbalance problem

## Abstract

Background: Although polysomnography (PSG) is a gold standard tool for diagnosing sleep apnea (SA), it can reduce the patient’s sleep quality by the placement of several disturbing sensors and can only be interpreted by a highly trained sleep technician or scientist. In recent years, electrocardiogram (ECG)-derived respiration (EDR) and heart rate variability (HRV) have been used to automatically diagnose SA and reduce the drawbacks of PSG. Up to now, most of the proposed approaches focus on machine-learning (ML) algorithms and feature engineering, which require prior expert knowledge and experience. The present study proposes an SA detection algorithm to differentiate a normal and apnea event using a deep-learning (DL) framework based on 1D and 2D deep CNN with empirical mode decomposition (EMD) of a preprocessed ECG signal. The EMD is ideally suited to extract essential components which are characteristic of the underlying biological or physiological processes. In addition, the simple and compact architecture of 1D deep CNN, which only performs 1D convolutions, and pretrained 2D deep CNNs, are suitable for real-time and low-cost hardware implementation. Method: This study was validated using 7 h to nearly 10 h overnight ECG recordings from 33 subjects with an average apnea-hypopnea index (AHI) of 30.23/h originated from PhysioNet Apnea-ECG database (PAED). In preprocessing, the raw ECG signal was normalized and filtered using the FIR band pass filter. The preprocessed ECG signal was then decomposed using the empirical mode decomposition (EMD) technique to generate several features. Several important generated features were selected using neighborhood component analysis (NCA). Finally, deep learning algorithm based on 1D and 2D deep CNN were used to perform the classification of normal and apnea event. The synthetic minority oversampling technique (SMOTE) was also applied to evaluate the influence of the imbalanced data problem. Results: The segment-level classification performance had 93.8% accuracy with 94.9% sensitivity and 92.7% specificity based on 5-fold cross-validation (5fold-CV), meanwhile, the subject-level classification performance had 83.5% accuracy with 75.9% sensitivity and 88.7% specificity based on leave-one-subject-out cross-validation (LOSO-CV). Conclusion: A novel and robust SA detection algorithm based on the ECG decomposed signal using EMD and deep CNN was successfully developed in this study.

## 1. Introduction

Sleep apnea (SA) is a common sleep disorder in which pauses in breathing or periods of shallow breathing during sleep occur more often than normal, which often remains undiagnosed and untreated [1]. Each pause possibly lasts between 10 and 20 s and happens from 5 to over 100 times per hour during overnight sleep. SA can present with or without symptoms and is accompanied by major neurocognitive and cardiovascular sequelae. SA is characterized by increased collapsibility of the upper airway during sleep, resulting in significantly reduced (hypopnea) or absent (apnea) airflow at the nose and/or mouth, which is usually accompanied by oxyhemoglobin desaturation and is typically terminated by a brief micro-arousal [2]. Repeated apnea episodes result in a prolonged decrease in oxyhemoglobin saturation and sleep fragmentation, with less slow-wave and rapid eye movement (REM) sleep.

SA has a high prevalence, especially among middle-aged and elderly people. According to the American Academy of Sleep Apnea’s diagnostic criteria, approximately 936 million adults aged 30 to 69 years have mild to severe SA syndrome and 425 million adults aged 30 to 69 years have moderate to severe SA (AASM) [3,4]. Further research into the relationship between the incidence of SA and age found that 88% of men aged 65 to 69 years had five or more events per hour, while that incidence increased to 90% in men aged 70 to 85 years. In addition, SA has been linked to an increased risk of other diseases such as hypertension, heart failure, a heart attack, and a cardiovascular event [5].

Among the causes, there is the use of different diagnostic tools. For AASM, the gold standard for a SA diagnosis remains comprehensive polysomnography [6], although alternative diagnosis methods have been studied. In recent decades, different SA detection methods have been proposed using a single-lead physiological signal to satisfy the non-intrusive demand and hardware conditions of wearable mobile equipment, such as pulse oximetry (SpO_2_), nasal airflow, thoracic (THO) and abdominal (ABD) movement electroencephalography (EEG), and electrocardiography (ECG). Based on SpO_2_ signal, Alvarez et al. built a home polysomnography-based SA screening system using machine learning [7], and Mendonca et al. designed a wireless home monitoring device for automatic diagnosis OSA patient [8]. Based on nasal airflow, Haidar et al. proposed an apnea-hypopnea events recognition system using convolutional neural network (CNN) [9] and Yue et al. developed a multi-resolution residual network for the diagnosis and classification of OSA [10]. Based on THO and ABD movement signals, Lin et al. identified various types of apneas using wearable piezoelectric bands [11]. Based on EEG signal, Zhao et al. developed an algorithm to automatically differentiate SA events using entropy-based EEG sub-band feature extraction and machine learning-based classifiers [12]. Bhattacharjee et al. aimed to detect SA based on Rician modeling of feature variation and in multi-band EEG [13,14], and Taran et al. introduced an apnea event detection algorithm using Hermite basis function adaptive decomposition for EEG [15].

ECG waveform analysis and heart rate variability (HRV) have recently been used and developed as an alternative method of detecting sleep disordered breathing, including apnea and hypopnea events [16,17]. The ECG signal is particularly interesting to study because it enables the physiological demonstration of SA occurrence and is easy to record with wearable devices. When an apnea event occurs, blood oxygen levels drop, and the cardiovascular system is prompted to keep the body’s oxygen supply adequate. As a result, abnormal heart activity or high heart rate variability may indicate the presence of SA. The respiratory effort produces an alteration in the ECG electrode’s position, which severally influences the ECG signal’s amplitude [18]. Meanwhile, the HRV assesses the time interval variation between successive heartbeats, known as the R-R interval (RRI). The variation in the RRI is a symptom of apnea events, hence can provide the physiological basis to detect SA. The possibility of diagnosing SA by single-lead ECG was presented in 1984 by Guilleminault et al. [19], followed by an apnea ECG PhysioNet database publication after competition called the Computers in Cardiology 2000 Challenge [16], which provided minute-by-minute ECG data with apnea labeling. In recent years, more studies using single-lead ECG to detect apnea episodes have been proposed and validated using the PhysioNet database. The majority of them concentrated on machine learning (ML) and feature engineering, which necessitates the use of a specific feature extraction method to extract ECG features. Otherwise, the deep learning approach has been developed in recent years. The SA detection literature review based on single-lead ECG using ML and DL frameworks is summarized in Table 1.

The novelty and significance of this study can mainly be described as follows:

(1) The proposed method used EMD features generated from preprocessed overnight ECG signals to identify various symptoms of SA episodes. Due to external stimulation, biomedical signals are, in general, non-linear and non-stationary, thus EMD is ideally suited to extract essential components which are characteristic of the underlying biological or physiological processes.
(2) The proposed deep learning framework can outperform SA classification state-of-the-art methods with potential for real-time and low-cost hardware implementation. The deep CNN models have a simple and compact architecture; moreover, they reduce the need for feature extraction.
(3) The proposed method applied the synthetic minority oversampling technique (SMOTE) to overcome the imbalance problem. The comparison performance with other re-sampling methods is also discussed.

The remaining focus of this paper is organized as follows. Section 2 describes the databases that were used as well as the proposed SA detection system that is based on EMD and deep CNN models. Section 3 contains the results of the experiments. Section 4 describes the study findings discussions. Finally, Section 5 brings the study to a close.

## 2. Materials and Methods

### 2.1. Overnight Apnea ECG Database

The Apnea ECG PhysioNet Database (AEPD) [40] is an open public database and was utilized to validate and enable the proposed algorithm performance to be evaluated and compared with the existing literature. The AEPD ECG recordings were sampled at 100 Hz, and the duration of each participant’s sleep recording ranged between 7 and 10 h. [16]. The database contained 35 participants’ recordings with SA annotations on a minute-by-minute basis; each minute of ECG recording was annotated as either normal breathing or disordered breathing with apnea episode.

AEPD had three participant categories: A: apnea (Table 2), B: borderline (Table 3), and C: healthy control (Table 4), each with 20, 5, and 10 subjects. Group A participants were discovered to be related to people with OSA and had total apnea durations of more than 100 min for each recording. This group’s participants ranged in age from 38 to 63, and their AHI ranged from 21 to 83. Group B participants were borderline, with total apnea episode durations ranging from 10–96 min. This group’s age ranged from 42 to 53, and the AHI ranged from 0 to 25. Group C participants were healthy controls with no SA or very low levels of disease and total apnea durations ranging from 0 to 3 min. Group B recording b05 and Group C recording c05 were eliminated because b05 contained a grinding noise and c05 was identical to c06. Consequently, there were only 33 recordings included in this study, with totally 9160 normal events and 6019 apnea events (imbalanced dataset), after eliminating some contaminated events caused by the patient’s movement, poor patch contact, electrical interference, measurement noise, or other disturbances. Table 2, Table 3 and Table 4 provide the details on each participant’s characteristics and the sample size information used in this study.

### 2.2. The Overall Deep Learning Framework for Automatic Sleep Apnea Classification Based on Empirical Mode Decomposition Derived from Single-Lead Electrocardiogram

**Figure 1:**
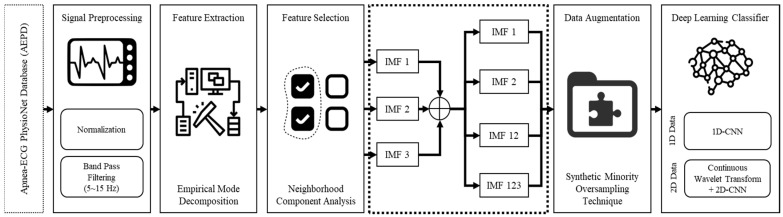
The proposed sleep apnea detection system flowchart based on the deep learning framework and EMD of single lead ECG signal.

Figure 1 shows the overall proposed sleep apnea detection system flowchart based on the deep learning framework and the ECG signal decomposition. This study proposes an automatic SA classification algorithm based on the deep learning framework and EMD derived from a single-lead ECG signal of apnea and normal breathing. Overnight single-lead ECG signals from 33 participants were the input with each participant’s 7~8 h recordings, and then partitioned into subsequent 60-s ECG windows. The participants of the mixed group category comprised AEPD group A, B, and C. The raw ECG signal was preprocessed and included normalization, windowing, and band-pass filtering. The EMD algorithm was used to obtain decomposition features to discriminate SA and normal breathing episodes. Feature selection based on the neighborhood component analysis (NCA) was then applied to reduce the number of EMD features (IMFs). In total, 4 IMFs were used in this study, specifically ${\mathrm{IMF}}_{1}$, ${\mathrm{IMF}}_{2}$, ${\mathrm{IMF}}_{12}$ as the result of the addition IMF_1_ and _2_ and ${\mathrm{IMF}}_{123}$ as the result of the addition IMF_1_, _2_, and _3_. Thereafter, the synthetic minority oversampling technique (SMOTE) was applied to deal with the data imbalance problem. Finally, a 1D deep CNN model was established to obtain the classification results using cross-validation.

### 2.3. ECG Signal Preprocessing and Filtering

The zero-means computation and windowing processing parameters were used in the ECG signal preprocessing. In this method, zero-means subtracts the mean from the ECG signals to remove the baseline wandering effects, and then nocturnal ECG spectra are divided into 60-s intervals, with large noise windows excluded for algorithm development. The AEPD used in this study labeled each 60-s window as having an apnea or normal episode.

### 2.4. Empirical Mode Decomposition (EMD)

Empirical mode decomposition (EMD) [41] is a time-frequency domain signal analysis that adaptively and locally decomposes any non-stationary time series in a sum of oscillatory waveforms known as Intrinsic Mode Functions (IMF), which represent zero-mean amplitude and frequency modulated components. The main idea of this method is to empirically identify these intrinsic oscillatory modes by their characteristic time scales in the data, and then decompose the data accordingly. The step by step of EMD generation is as follows:

1. Correlate all the local maximum and minimum by applying a cubic spline to generate the upper and lower envelope.
2. Specify the average of upper and lower envelopes, which is denoted as $m_{1}\left(t\right)$.
3. Calculate the difference between the signal y(t) and the average $m_{1}\left(t\right)$, $i_{1}\left(t\right)=y\left(t\right)-m_{1}\left(t\right)$ (potential first IMF).
4. Verify if $i_{1}\left(t\right)$ satisfies the two requirements of IMF:
  - In the entire data set, the number of extremum and zero-crossings must be equal or differ by no more than one.
  - The mean value of the envelope indicated by the local maximum and local minimum is zero at any point.

If $i_{1}\left(t\right)$ meets both requirements, then $i_{1}\left(t\right)$ is the first IMF of the y(t) signal. If $i_{1}\left(t\right)$ does not meet the two IMF requirements, the sifting process will be reiterated by treating the $i_{1}\left(t\right)$ as original until it meets the requirements.

5. After subtracting the y(t) signal from the IMF, the sifting process is reiterated to break down the data into n IMFs.

**Figure 2:**
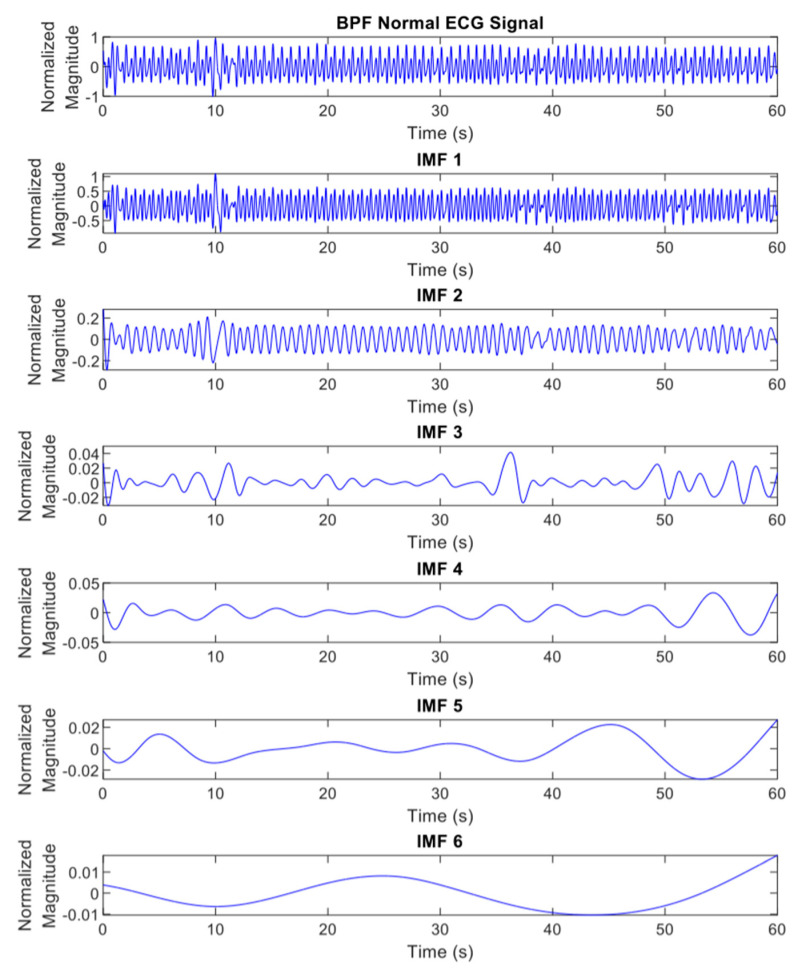
The EMD results (IMF_1_ to _6_) of preprocessed ECG signals of normal episode.

**Figure 3:**
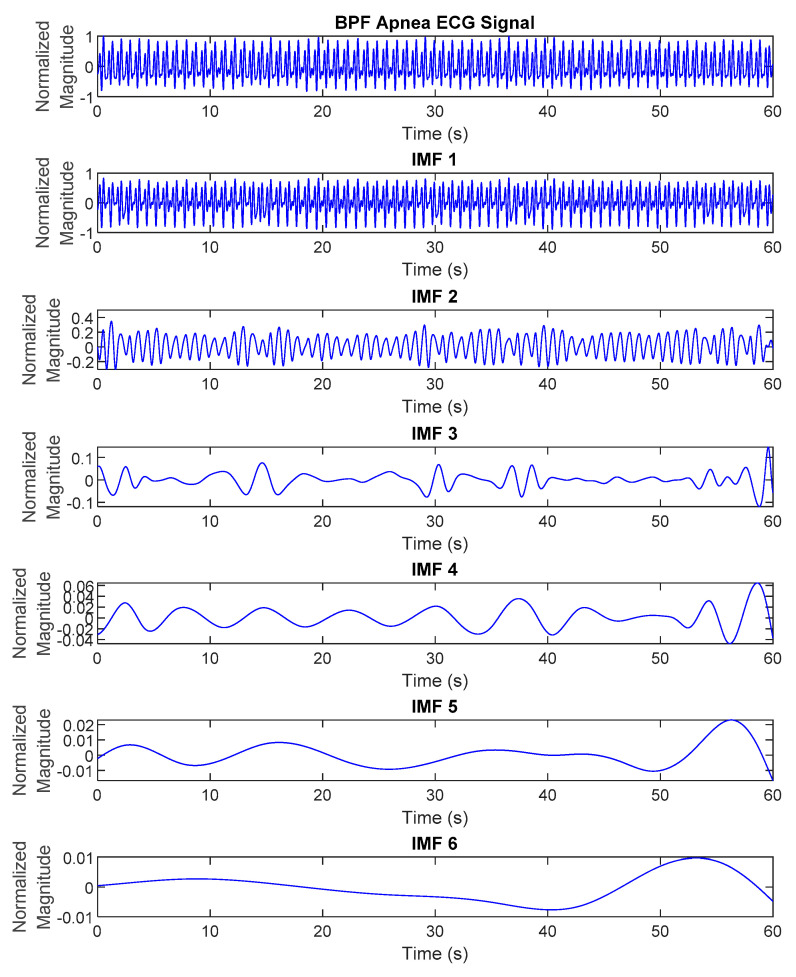
The EMD results (IMF_1_ to _6_) of preprocessed ECG signals of apnea episode.

Finally, the signal y(t) can be expressed as:

$$y\left(t\right)=\overset{n}{\underset{j=1}{\sum }}i_{j}\left(t\right)+r_{n}\left(t\right)$$

where $i_{j}\left(t\right)$ represents the IMFs of the original signal y(t) with j=1,2,3,…,n and $r_{n}\left(t\right)$ is a residue of y(t). The differentiation of ECG decomposed signals, IMF_1_ to _6_, between normal and apnea episodes is clearly described in Figure 2 and Figure 3.

### 2.5. Feature Transformation Using Continuous Wavelet Transform (CWT)

The continuous wavelet transform (CWT) [42] is a technique for extracting time-localized information from a signal by calculating the time convolution of the signal with the analyzing wavelet. Convolution between the signal x(t) and a family (set) of wavelets ${\tilde{\psi }}_{f}\left(t\right)$ defined for a specific frequency range is required to compute the spectro-temporal representation of a signal using CWT, assigned as:

$$X\left(t,f\right)=x\left(t\right)*{\tilde{\psi }}_{f}\left(t\right)$$

**Figure 4:**
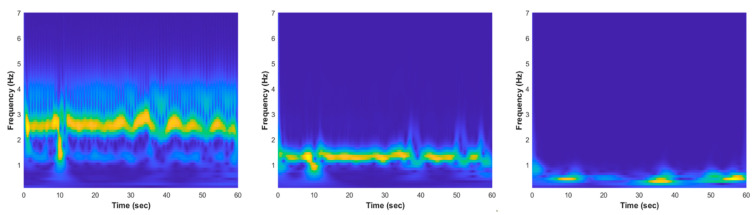
The EMD CWT spectrogram results of normal episode preprocessed ECG signal, from left to right: ${\mathrm{IMF}}_{1}$, ${\mathrm{IMF}}_{2}$, and ${\mathrm{IMF}}_{3}$.

**Figure 5:**
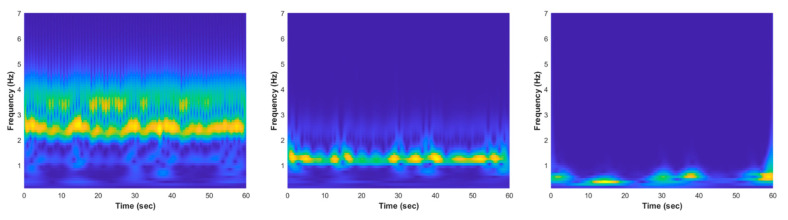
The EMD CWT spectrogram results of apnea episode preprocessed ECG signal, from left to right: ${\mathrm{IMF}}_{1}$, ${\mathrm{IMF}}_{2}$, and ${\mathrm{IMF}}_{3}$.

To extract amplitude and phase, wavelet functions must be complex-valued and well-localized in time and frequency. The complex Morlet wavelet is the most commonly used wavelet for complex spectro-temporal signal characterization. It is made up of a complex oscillation with a fixed frequency (frequency localization) that is constrained by a Gaussian window (time localization). The mother wavelet for the complex Morlet wavelet family is formulated as:

$${\tilde{\psi }}_{f_{0}}\left(t\right)=A\left(\sigma_{t}\right)e^{-t^{2}/2\sigma_{t}^{2}}e^{j2\pi f_{0}t}$$

$$\mathrm{with}\;A\left(\sigma_{t}\right)=\frac{1}{\sqrt{\sigma_{t}\sqrt{\pi }}}\;\mathrm{and}\;\sigma_{t}\left(f_{0}\right)=\frac{n_{c}}{2\pi f_{0}}$$

where $e^{j2\pi f_{0}t}$ is the complex oscillatory component with frequency of $f_{0}$ Hz, $e^{-t^{2}/2\sigma_{t}^{2}}$ is the Gaussian window with a temporal standard deviation of $\sigma_{t}$, $A\left(\sigma_{t}\right)$ is applied to confirm that the wavelet energy is equal to one, and the parameter $n_{c}$ generally specifies the number of cycles at the frequency $f_{0}$ inside the Gaussian bell. The CWT spectrograms of normal and apnea episode from the EMD preprocessed ECG signal are clearly presented in Figure 4 and Figure 5.

### 2.6. Feature Selection Using Neighborhood Component Analysis (NCA)

Feature selection is the process of reducing the number of input variables when developing a predictive model. It is desirable to reduce the number of input variables to both reduce the computational cost of modelling and, in some cases, to improve the performance of the model. Neighborhood component analysis (NCA) is a non-parametric method for selecting features with the goal of maximizing prediction accuracy of classification algorithms [43].

The regularization parameter (λ) was tuned for the best features. NCA discards irrelevant features which reduces the features dimensionality and improves the algorithm performance. The steps involved in NCA are as follows [44]:

- Data is divided into training and testing sets. The training data are then partitioned into 10-folds in which the classifier leaves out one-fold for testing and trained on the other nine folds.
- The regularization parameter, λ value, is tuned and the NCA model was trained for each λ using each fold in the training set. This process is repeated for all folds and all λ values.
- The average loss obtained from each fold for each λ value was computed and the best λ value was found, which corresponds to the minimum average loss.
- Finally, the features with feature weight greater than the threshold (*T*) were extracted.

T=τ×max(w)

where, τ is tolerance fixed to 0.02 and w is updated features weight.

As the result, the selected EMD features were IMF_1_, _2_, and _3_ based on the NCA evaluation of mean, standard deviation, and variance of each feature. The selected IMFs were also combined and generated new features, such as ${\mathrm{IMF}}_{12}$ and ${\mathrm{IMF}}_{123}$, to remove most of the noises and artifacts and construct denoised ECG signals. The combination IMFs were defined as follows:

$${\mathrm{IMF}}_{12}=i_{1}\left(t\right)+i_{2}\left(t\right)$$

$${\mathrm{IMF}}_{123}=i_{1}\left(t\right)+i_{2}\left(t\right)+i_{3}\left(t\right)$$

**Figure 6:**
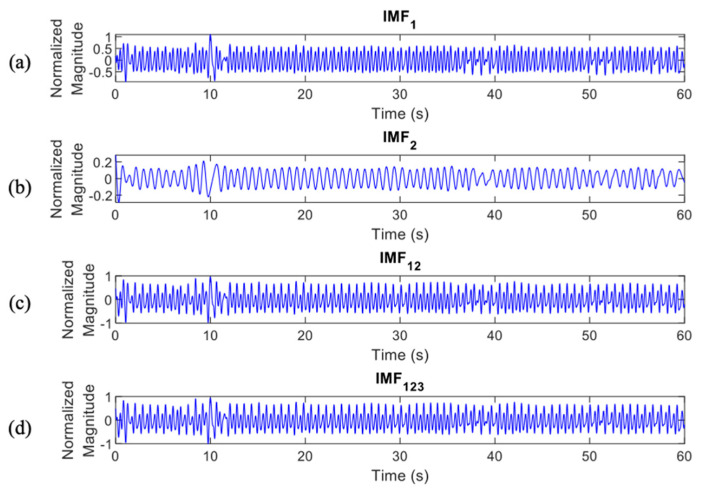
IMF features of normal episodes which were processed to the classification stage, ${\mathrm{IMF}}_{1}$ (**a**), ${\mathrm{IMF}}_{2}$ (**b**), ${\mathrm{IMF}}_{12}$ (**c**), and ${\mathrm{IMF}}_{123}$ (**d**).

**Figure 7:**
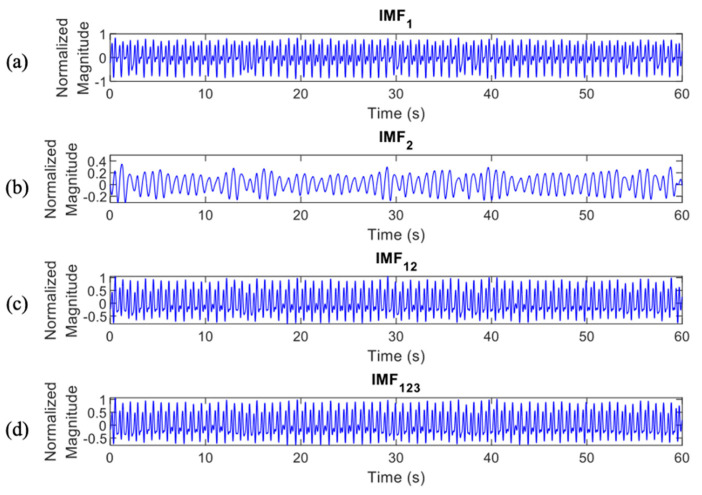
IMF features of apnea episodes which were processed to the classification stage, ${\mathrm{IMF}}_{1}$ (**a**), ${\mathrm{IMF}}_{2}$ (**b**), ${\mathrm{IMF}}_{12}$ (**c**), and ${\mathrm{IMF}}_{123}$ (**d**).

In total, there were 4 IMF features, ${\mathrm{IMF}}_{1}$, ${\mathrm{IMF}}_{2}$, ${\mathrm{IMF}}_{12}$, and ${\mathrm{IMF}}_{123}$, which were processed to the classification stage (Figure 6 and Figure 7 provide details of the IMF features between normal and apnea episodes).

### 2.7. Data Augmentation Using Synthetic Minority Oversampling Technique (SMOTE)

The SMOTE algorithm [45] generates artificial examples based on the feature space, rather than data space, similarities between existing minority examples, by the reason of data augmentation. These synthetic examples are generated by connecting a portion or all of the minority class’s *K* nearest neighbors. Neighbors from the *K* nearest neighbors are chosen at random depending on the amount of oversampling required.

In more detail, let $S_{min}\;\epsilon \;S$ characterize the class with minority condition. For each data point $x_{i}\;\epsilon \;S_{min}$ find the *k*-nearest neighbor, given a specified *K*. The *k*-nearest neighbors are defined as the *K* elements of $S_{min}$ whose Euclidian distance between itself and $x_{i}$ have the smallest magnitude in the feature space *X*. To generate a new data point, choose one of the k-nearest neighbors at random, and then calculate the difference between the chosen data point and its nearest data point. Multiply this difference by a number *λ*, that ranges from 0 to 1. Finally, add this vector to the selected data point $x_{i}$:

$$x_{syn}=x_{i}+\left({\hat{x}}_{i}-x_{i}\right)\times \lambda$$

where $x_{i}\;\epsilon \;S_{min}$ is the selected instance from minority class, ${\hat{x}}_{i}\;\epsilon \;S_{min}$ is one of the k-nearest neighbors of $x_{i}$ and λ ϵ [0,1] is random generated number.

### 2.8. One-Dimensional Deep Convolutional Neural Network (1D Deep CNN)

Deep-learning (DL) is the most recent achievement of the machine-learning (ML) era, displaying near-human [46], and now super-human abilities in a variety of applications such as voice-to-text translations, object detection and recognition, anomaly detection, emotion recognition from audio or video recordings, and so on.

**Figure 8:**
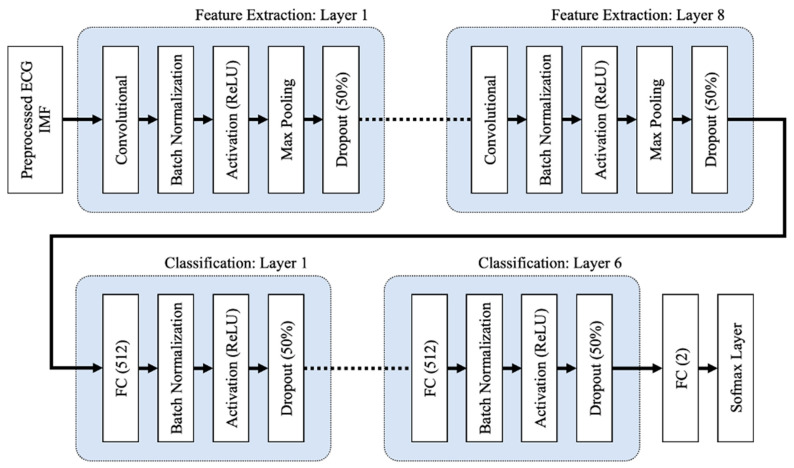
The 1D deep CNN architecture, comprises of input, feature extraction, and classification stages.

The proposed 1D deep CNN architecture, adapted from a study by Chang et al. [25], comprises input, feature extraction, classification, and output stages, as shown in Figure 8 and Table 5.

To initialize the weights, the CNN and FC layers both use the He normal initialization method. The weights are initialized with the previous layer of neurons in mind, which helps the cost function reach the global minimum faster and more efficiently. The batch normalization layers, which are added after the CNN and FC layers in both the feature extraction and classification layers, normalize the data before it enters the ReLU activation layer, which improves the neural network’s speed, performance, and stability. By selecting the maximum activation about a neuron in a feature map, the max pooling layers in the feature extraction layers reduce network complexity and the possibility of overfitting. When the pooling size is set to 2, the size of each feature map is cut in half. Dropout layers with a dropout rate of 0.5 are used to reduce overfitting by randomly omitting 50% of the nodes during the proposed CNN model’s training process. Overfitting results in a high training accuracy but a low testing accuracy. The proposed 1D deep CNN model was trained to minimize cross entropy using the Adam optimizer, which is a stochastic gradient descent extension that computes individual adaptive learning rates for different parameters based on estimates of the gradient’s first and second moments.

### 2.9. Two-Dimensional Deep Convolutional Neural Network (2D Deep CNN)

Transfer learning is the enhancement of learning in a new task by the transfer of knowledge from a previously learned network. Fine-tuning with transfer learning is typically considerably faster and easier than training a network with randomly initialized weights from beginning. There were three kinds of pretrained CNN models that were employed for transfer learning in this study, i.e., AlexNet, GoogLeNet, and ResNet-50. Table 6 explains the main differences among the pretrained CNN models, AlexNet, GoogLeNet, and ResNet-50.

AlexNet [47] architecture consists of 8 layers, including 5 convolution layers (with 2 Convolution 2D layers, 3 Grouped Convolution 2D layers, 5 Rectified Linear Unit (ReLU) layers, 2 Cross-Channel Normalization layers, 3 Max-Pooling 2D layers) and 3 fully connected layers (with 3 Fully Connected layer, 2 ReLU layers, 2 Dropout layers for regularization, a Softmax layer using a normalized exponential function). The network’s originality is the effective implementation of the ReLU activation function, as well as the usage of the Dropout mechanism and data augmentation technique to prevent overfitting. A Cross-Channel Normalization layer is used in the network to increase model generalization. Furthermore, maximum overlap pooling is employed to eliminate the blurring effect generated by average pooling.

GoogLeNet [48] is a pretrained CNN that has 22 layers with 9 inception layers. In a convolutional vision network, an inception layer determines the optimal local sparse structure, which can be approximated and covered by readily available dense components. The network includes an initial structure to expand the network’s width and depth while eliminating the fully connected layer and replacing it with average pooling to prevent the gradient disappearance.

ResNet-50 [49] denotes a residual network (ResNet) built with 50 layers. The central concept of a ResNet is the presentation of an “identity shortcut connection” that bypasses one or more layers. A shortcut (or skip) connection is used to solve the problem of vanishing or exploding gradients by re-routing the input and adding to the previous layer’s concept. A layer learns the concepts of the previous layer and merges with inputs from that previous layer during learning.

### 2.10. Cross-Validation

Cross-validation is a statistical method for evaluating and comparing learning algorithms that divides data into two groups: a training set (for model training) and a testing set (for model testing or validation) [50]. The training and testing sets must cross over in successive rounds so that each data point can be validated. Cross-validation is used for two main reasons: First, the performance of the learned model from available data can be investigated using a single algorithm. In other words, it is used to assess an algorithm’s generalizability. The second goal is to compare the performance of two or more different algorithms to find the best algorithm for the available data or, alternatively, to compare the performance of two or more parameterized model variants. Leave-one-subject-out cross-validation (LOSO-CV) and k-fold cross-validation (*k*fold-CV) were performed at segment-level and subject-level validation, respectively.

**Figure 9:**
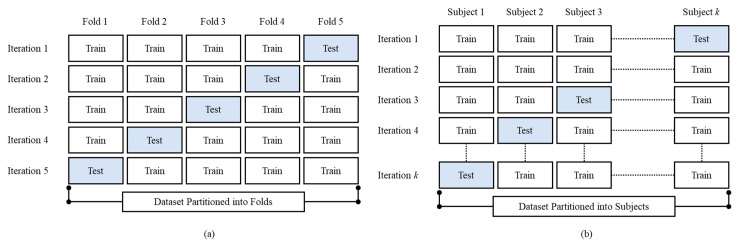
The proposed method cross-validation illustration, (**a**) *k*fold-CV (k = 5) and (**b**) LOSO-CV.

In the *k*fold-CV (Figure 9a), the first step is to a create division of the data samples randomly into *k* subgroups. Afterward, each subgroup can be chosen as the testing set with the remaining (*k*-1) subgroups as the training set. In this manner, *k*fold-CV repeats training and testing *k* times, and the final accuracy is the average of the *k* accuracy values for each iteration. Meanwhile, in the LOSO-CV, the records of only one subject are excluded for each training process. In other words, consider N the number of people in the dataset, training is finished on N-1 subjects and validation is finished on a single subject. This process is repeated N times (Figure 9b).

## 3. Experimental Results

This study was conducted using MATLAB 2021b software (developed by MathWorks, Natick, MA, USA) on several computers with 24 GB installed RAM, Intel^®^ Core^TM^ i5-8400 CPU ≅ 2.80 GHz, and NVIDIA GeForce GTX 1060 6 GB installed graphic card. The commonly used performance evaluation parameters for the apnea detection system are defined as follows:

$$\mathrm{Accuracy}=\frac{\mathrm{TP}+\mathrm{TN}}{\mathrm{TP}+\mathrm{FP}+\mathrm{TN}+\mathrm{FN}}\times 100\%$$

$$\mathrm{Sensitivity}=\frac{\mathrm{TP}}{\mathrm{TP}+\mathrm{FN}}\times 100\%$$

$$\mathrm{Specificity}=\frac{\mathrm{TN}}{\mathrm{TN}+\mathrm{FP}}\times 100\%$$

where TP indicates the number of positive events predicted correctly (apnea events predicted as apnea events), FP indicates the number of actual negative events (normal events) predicted as positive events (apnea events), TN indicates the number of negative events predicted correctly (normal events predicted as normal events), FN indicates the number of actual positive events (apnea events) predicted as negative events (normal events). The receiver operating characteristic (ROC) then plotted and computed the area under the ROC curve (AUC) to globally specify the apnea detection performance [51].

Youden’s index is commonly used to assess the effectiveness of an overall diagnostic test when deciding between two or more diagnostic tests [52]. In other words, this index was used to choose the best classification result from a variety of input systems, such as dataset setting and feature. Youden’s index is a function of sensitivity and specificity and ranges from 0 to 1, with a value close to 1 indicating that the diagnostic test is meaningful and a value close to 0 indicating that the diagnostic test is useless. The Youden’s index (J) is calculated as the sum of the measurement correctly diagnosed for the diseased group (sensitivity and healthy group (specificity).

J=(sensitivity+specificity)−1

### 3.1. Per Segment Classification

**Figure 10:**
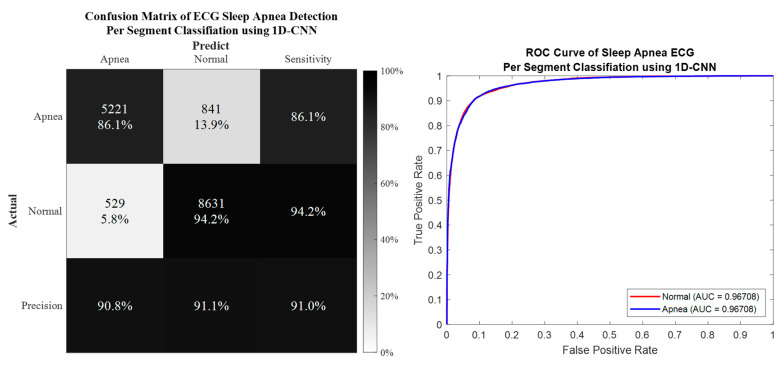
The confusion matrix and ROC curve of the best 1D deep CNN 5fold-CV performance of imbalanced dataset.

**Figure 11:**
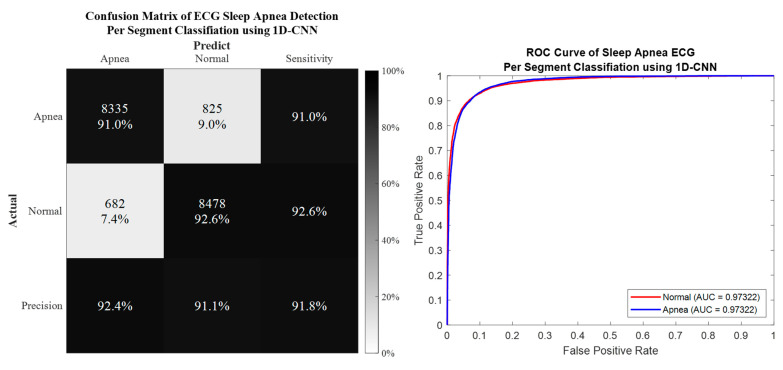
The confusion matrix and ROC curve of the best 1D deep CNN 5fold-CV performance of SMOTE dataset.

**Figure 12:**
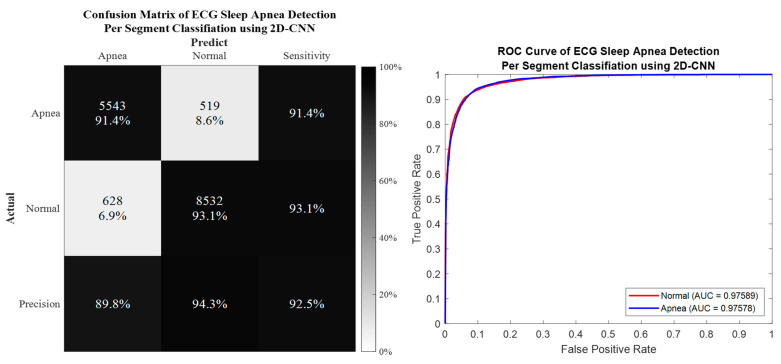
The confusion matrix and ROC curve of the best 2D deep CNN (ResNet-50) 5fold-CV of imbalanced dataset.

**Figure 13:**
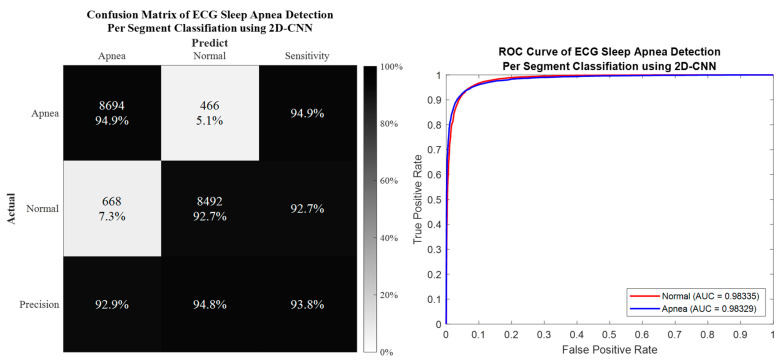
The confusion matrix and ROC curve of the best 2D deep CNN (ResNet-50) 5fold-CV of SMOTE dataset.

Per segment classification was performed using 5-fold cross-validation (5fold-CV). The detailed classification results are given in Table 7 and Figure 10, Figure 11, Figure 12 and Figure 13. The best per segment classification performance of mixed group SA imbalanced dataset was from ${\mathrm{IMF}}_{12}$ of ResNet-50 with 92.5% accuracy, 91.4% sensitivity, 93.1% specificity, and 0.9758 AUC value. The best per segment classification performance of mixed group SA balanced dataset based on SMOTE was from ${\mathrm{IMF}}_{123}$ with 93.8% accuracy, 94.9% sensitivity, 92.7% specificity, and 0.9833 of AUC value.

### 3.2. Per Subject Classification

A more applicable approach is so-called per-subject classification, which is required to test a new unseen participant into the trained model. Per-subject classification was performed using leave-one-subject-out cross-validation (LOSO-CV). The detailed classification results are given in Table 8, Table 9, Table 10 and Table 11. The best per subject classification performance of mixed group SA was from ${\mathrm{IMF}}_{12}$ ResNet-50 with 83.5% accuracy, 75.9% sensitivity, and 88.7% specificity.

## 4. Discussion

### 4.1. Performance Comparison between Proposed Method and Existing Literature

Table 12 compares the proposed SA detection algorithm with several latest best-practiced DL and ML algorithms from the existing literature using AEPD. In recent years, a DL framework in SA classification has been developed. Qin et al. [21] proposed a dual-model deep learning method to perform representation learning and long-term temporal dependance with the adaptive synthetic (ADSYN) sampling method based on the R-R interval to classify a SA event (91.1% accuracy, 88.9% sensitivity, and 92.4% specificity). Yeh et al. [22] developed filter bank decomposition to decompose the ECG signal and a 1D-CNN to extract and classify each sub band decomposed ECG signal (88.6% accuracy, 83.8% sensitivity, and 91.5% specificity). Feng et al. [23] developed a SA detection model based on frequential stacked sparse auto-encoder (FSSAE) and time-dependent cost-sensitive (TDCS) classification (85.1% accuracy, 86.2% sensitivity, and 84.4% specificity). Sheta et al. [24] proposed an automated computer-aided diagnosis system to diagnose apnea events based on ECG using deep learning models (86.3% accuracy and 88.8% sensitivity). Chang et al. [25] developed a 1D deep CNN model using single-lead ECG recordings to construct a sleep apnea detection system (87.9% accuracy, 92% sensitivity, 81.1% specificity). Singh et al. [30] proposed a CNN-based deep learning approach using the time-frequency scalogram transformation of an ECG signal (86.22% accuracy, 90% sensitivity, and 83.8% specificity). Li et al. [53] proposed a SA detection system using a deep neural network-based and Hidden Markov model (HMM) based on single-lead ECG (84.7% accuracy, 88.9 sensitivity, and 82.1 specificity).

The ML framework development for SA classification is very dependable with feature engineering. Lin et al. [20] developed a SA detection method using the ML framework and bag-of-features derived from an ECG spectrogram (91.4% accuracy, 89.8% sensitivity, and 92.4 specificity). Bozkurt et al. [27] aimed to develop new machine learning-based respiratory scoring in the SA diagnostic process with feature engineering of HRV and ECG signal (85.1% accuracy, 85% sensitivity, 86% specificity). Surrel et al. [34] used the R-R intervals and R-S amplitude series with apnea scoring energy feature generation and SVM (85.70% accuracy, 81.40% sensitivity, and 88.40% specificity). Hassan et al. [35] addressed the automated sleep apnea detection system using a data-adaptive signal decomposition tunable-Q factor wavelet transform (TQWT) for single-lead ECG and a random under sampling boosting (RUSBoost) classifier (88.9% accuracy, 87.6% sensitivity, and 91.5% specificity). The proposed method achieved significantly better performance in accuracy, sensitivity, specificity, and AUC compared to all other considered methods.

### 4.2. Dealing with Imbalanced Dataset Problem

The imbalanced dataset problem exists when an uneven distribution of elements across the decision classes occurs: one class of data (typically the diseased class) is smaller than the other classes. In the study of SA classification, most events are normal and only a minority of apnea events occur, since not only participants with apnea were included but also the borderline and healthy control participants. This problem causes a condition where the trained classifier’s performance for normal events data, expressed as the specificity, is much higher than the sensitivity. The sensitivity metric represents the ability of the classifier to recognize patients (apnea events class) and if a classifier is only accurate for normal people, it is not valuable. To overcome this problem, certain synthetic data balancing methods need to be applied to balance the dataset (see Table 13 for some existing research in dealing with imbalanced data of SA classification).

The main objective of balancing classes is to either increase the frequency of the minority class or decrease the frequency of the majority class by re-sampling techniques. Random over sampling (ROS) aims to increase the number of instances in the minority class by randomly replicating them to present a higher representation of the minority class in the sample. Random under sampling (RUS) balances class distribution by randomly eliminating majority class examples. Table 6 presents the mixed group SA classification performance comparison using AEPD for several balancing methods, including ROS, RUS, and SMOTE (the proposed method).

As shown in Table 14, all methods could improve the SA classification performance, especially the sensitivity, which was much closer to the specificity. SMOTE and ROS could achieve superior performance and RUS had a slightly lower performance for all IMF features. Even though the best classification performance was also achieved by the ROS, SMOTE is still favored. This method can mitigate the problem of overfitting caused by ROS as synthetic examples are generated rather than replication of instances.

### 4.3. Intrinsic Mode Decomposition Feature Phenomenon of Apnea ECG Signal

Many studies have described the ECG signal to be highly correlated with respiratory variation [38,56,58,59]. During apnea onset, some key features can particularly be observed on the ECG signal, such as a decline in heart rate and an increase in heart rate followed by the end of the apnea episode. By decomposing the ECG signal using EMD, these key features are enhanced in certain IMFs. EMD deconstructs a signal into its key components (IMFs), which may then be used in evidence-specific dynamics to provide a more complete description of the signal [41,60]. EMD directly extracts energy connected with distinct intrinsic time scales.

**Figure 14:**
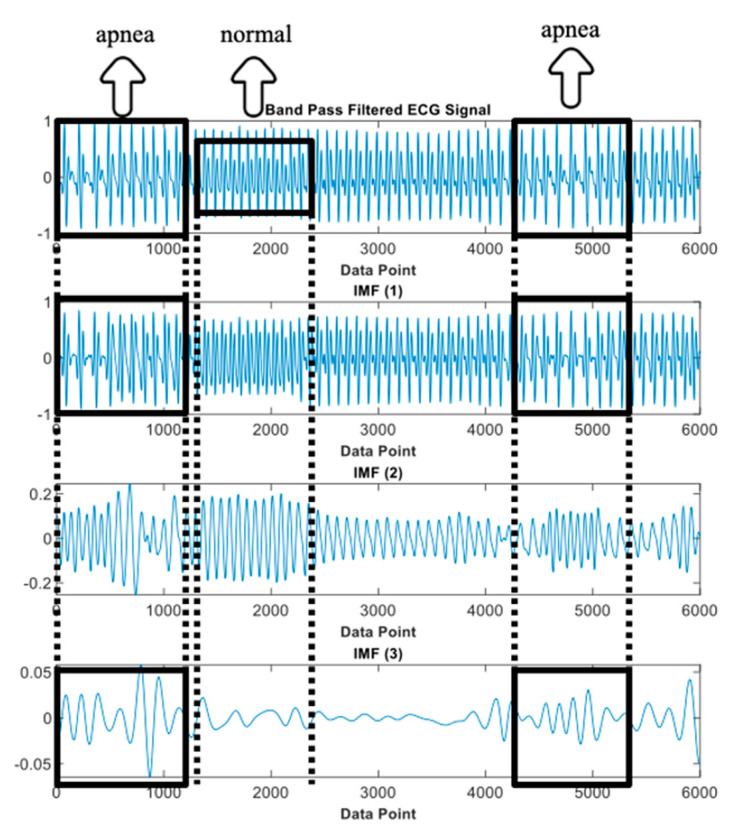
IMFs of preprocessed ECG observation (IMF_1_, IMF_2_, and IMF_3_).

The key features phenomenon of the apnea IMF ECG signal is clearly shown in Figure 14. This observation was only performed on three IMFs of the preprocessed apnea ECG (${\mathrm{IMF}}_{1}$, ${\mathrm{IMF}}_{2}$, and ${\mathrm{IMF}}_{3}$) since these IMFs were also the selected features input of the proposed method. In the normal event, all IMFs showed almost stable magnitude and frequency (relatively high); this also indicates that the heart rate does not change. Meanwhile, in the apnea event, the key features of heart rate were obviously emphasized. The apnea onset frequency of preprocessed ECG and ${\mathrm{IMF}}_{1}$ were not stable (tended to be higher in the end of episode) and lower than the apnea offset. Furthermore, on the ${\mathrm{IMF}}_{2}$ and ${\mathrm{IMF}}_{3}$, there were some explicit magnitude and frequency fluctuations.

The key features phenomenon differences between normal and apnea IMF ECG are emphasized and also possible to be observed from the CWT time-frequency spectrogram patterns visualization, as shown in Figure 4 and Figure 5. In the normal ${\mathrm{IMF}}_{1}$ ECG spectrogram, the dominant frequency range (yellow color region) was approximately 2–3 Hz, whereas in the apnea ${\mathrm{IMF}}_{1}$ ECG spectrogram, the dominant frequency range was around 2–4 Hz with unstable frequency power from 3 to 4 Hz. Additionally, the dominant frequency range of normal ${\mathrm{IMF}}_{2}$ ECG spectrogram was 1–2 Hz and apnea ${\mathrm{IMF}}_{2}$ ECG spectrogram was 1–3 Hz with lower frequency power in 2–3 Hz (light blue color region). These two spectrogram patterns indicate that the apnea episode had unstable frequency components compared to the normal episode.

### 4.4. Study Limitations and Future Developments

Despite the fact that the proposed algorithm performed exceptionally well, this study has several limitations. First, the proposed method was validated using a small sample size from AEPD. Second, insufficient physiological data and subject disease history were available for further analysis because AEPD did not provide such information. More data collection from the NCKUH Sleep Center, as well as the combination of multiple databases, may be a solution to these drawbacks. Third, the proposed algorithm could not be applied to patients with cardiovascular disease (CVD) complications because their ECG signal was somewhat irregular and not only affected by SA.

This study’s future developments include using explainable AI (XAI) to automatically identify physiological meanings for OSA features, testing the proposed method’s robustness in a large-scale group of participants from several databases, and developing an algorithm to discriminate SA and CVD using ECG data.

## 5. Conclusions

This paper proposed a novel algorithm to classify SA and normal breathing episodes using the signal decomposition method. Four different EMD features were considered along with 1D and 2D deep learning classifiers. When the ECG signal of SA and normal breathing episodes was decomposed, high accuracy was obtained. In addition, the algorithm outperformed the current best-practiced approaches in terms of accuracy.

By decomposing the ECG signal using EMD, the key features of normal and apnea episode are enhanced in certain IMFs. Furthermore, transforming the IMFs into the time-frequency spectrogram using CWT, pattern visualization and recognition of the key features made it possible to successfully differentiate the normal and apnea episodes.

## Figures and Tables

**Table 1:** Literature review of SA detection method based on single-lead ECG signal.

Author (Year)	Framework	Method	
		ECG Feature	Classifier
Lin et al. (2022) [20]	ML	Raw ECG	CWT + BoF + SVM/KNN/EL
Qin et al. (2022) [21]	DL	RRI	1D-CNN + BiGRU
Yeh et al. (2022) [22]	DL	Preprocessed ECG	Filter Bank Decomposition + 1D-CNN
Feng et al. (2021) [23]	DL	RRI	Stacked SAE + TDCS ^1^
Sheta et al. (2021) [24]	DL	Preprocessed ECG	CNN + LSTM
Chang et al. (2020) [25]	DL	Preprocessed ECG	1D-CNN
Zarei et al. (2020) [26]	ML	HRV and EDR	Feature Engineering ^2^ + ML Classifiers
Bozkurt et al. (2020) [27]	ML	HRV and ECG	Feature Engineering ^2^ + Fisher + EL
Viswabhargav et al. (2019) [28]	ML	RRI and EDR	SRE + SVM
Zarei et al. (2019) [29]	ML	Preprocessed ECG	DWT + Feature Engineering ^2^ + ML Classifiers
Singh et al. (2019) [30]	DL	ECG	CWT + CNN + SVM
Li et al. (2018) [31]	DL	RRI	Stacked SAE + Decision Fusion ^3^
Sharma et al. (2018) [32]	ML	Preprocessed ECG	BAWFB + FE and LE Features + LS-SVM
Tripathy (2018) [33]	ML	HRV and EDR	FDM + FE and Energy Features + KELM
Surrel et al. (2018) [34]	ML	RRI and RSA	Apnea Scoring (Energy) + SVM
Hassan et al. (2017) [35]	ML	Raw ECG	TQWT + RUSBoost
Sharma et al. (2016) [36]	ML	QRS and RRI	Hermite basis function + ML Classifiers
Song et al. (2016) [37]	ML	RRI and EDR	HMM + SVM/LR/LDA/KNN
Varon et al. (2015) [38]	ML	EDR	Feature Engineering ^2^ + LDA/SVM/LS-SVM
Hassan (2015) [39]	ML	Raw ECG	EMD + Feature Engineering ^2^ + ELM

Note: ^1^ TDCS consisted of MetaCost model and Softmax-HMM. ^2^ Feature engineering consisted of statistical, entropy, or non-liner features generation. ^3^ Decision fusion was the combination of parameter estimation SVM and ANN with HMM. DL: deep learning; ML: machine learning; RRI: R-R interval; HRV: heart rate variability; EDR: ECG-derived respiration; RSA: R-S amplitude; QRS: QRS complex; EMD: empirical mode decomposition; CWT: continuous wavelet transform; BoF: bag of feature; SVM: support vector machine; KNN: k-nearest-neighbor; EL: ensemble learning; CNN: convolutional neural network; BiGRU: bidirectional gated recurrent unit; SAE: sparse autoencoder; TDCS: time-dependent cost-sensitive; LSTM: long-short term memory; RUSBoost: random under sampling boosting; ANN: artificial neural network; SRE: sparse residual entropy; DWT: discrete wavelet transform; BAWFB: biorthogonal antisymmetric wavelet filter bank; FE: fuzzy entropy; LE: log energy; LS-SVM: least-square support vector machine; FDM: Fourier decomposition method; KELM: kernel extreme learning machine; TQWT: tunable-Q factor wavelet transform; HMM: Hidden Markov Model; LR: linear regression; LDA: linear discriminant analysis; EMD: empirical mode decomposition; ELM: extreme learning machine.

**Table 2:** Apnea ECG PhysioNet Database (APED) apnea participants description.

ID	Age (Year)	Gender	Weight (kg)	Height (cm)	AHI	A (min)	N (min)
a01	51	M	102	175	69.6	461	19
a02	38	M	120	180	69.5	381	99
a03	54	M	80	168	39.1	222	258
a04	52	M	121	173	77.4	441	39
a05	58	M	78	176	41	244	176
a06	63	M	104	179	24.7	183	297
a07	44	M	105	177	63	294	186
a08	51	M	88	179	42	187	293
a09	52	M	82	178	31.7	366	114
a10	58	M	78	176	21	83	397
a11	58	M	103	168	14	182	238
a12	52	M	121	173	80.2	439	41
a13	51	M	88	179	42	235	245
a14	51	M	102	175	54.7	383	97
a15	60	M	113	176	52	356	124
a16	44	M	105	177	41	320	160
a17	40	M	96	179	33	138	222
a18	52	M	82	178	82.4	429	51
a19	55	M	90	178	34	185	295
a20	58	M	78	176	41	295	185
**Total Event**						5824	3536

Note: M: male; F: female; A: apnea event; N: normal event.

**Table 3:** Apnea ECG PhysioNet Database (APED) borderline participants description.

ID	Age (Year)	Gender	Weight (kg)	Height (cm)	AHI	A (Min)	N (Min)
b01	44	F	63	170	0.24	19	461
b02	53	M	85	176	19	93	387
b03	53	M	85	176	24	65	355
b04	42	M	64	180	0.7	10	410
**Total Event**						187	1613

Note: M: male; F: female; A: apnea event; N: normal event.

**Table 4:** Apnea ECG PhysioNet Database (APED) healthy control participants description.

ID	Age (Year)	Gender	Weight (kg)	Height (cm)	AHI	A (Min)	N (Min)
c01	31	M	74	184	0	0	480
c02	37	M	83	180	1	0	479
c03	39	M	65	184	0	0	420
c04	41	F	65	180	0	0	480
c06	28	F	65	171	0.25	1	419
c07	30	F	56	168	0	4	416
c08	42	M	64	180	0	0	480
c09	37	M	83	180	0	2	418
c10	27	M	72	184	0	1	419
**Total Event**						6	4011

Note: M: male; F: female; A: apnea event; N: normal event.

**Table 5:** The detail layer’s description summary of the proposed 1D deep CNN model.

Layers	Parameters	Output Size (1D)
Input		[6000, 1]
**Feature Extraction Layer 1**		
Convolution	filters = 45, kernel size = 32 padding = ‘same’ kernel initializer = ‘he_normal’	[6000, 45]
Batch Normalization		[6000, 45]
Activation	ReLU	[6000, 45]
Max Pooling	pool size = 2, strides = 2	[3000, 45]
Dropout	dropout rate = 0.5	[3000, 45]
…		
**Feature Extraction Layer 8**		
Convolution	filters = 45, kernel size = 32 padding = ‘same’ kernel initializer = ‘he_normal’	[46, 45]
Batch Normalization		[46, 45]
Activation	ReLU	[46, 45]
Max Pooling	pool size = 2, strides = 2	[23, 45]
Dropout	dropout rate = 0.5	[23, 45]
**Classification Layer 1**		
Fully Connected	units = 512 kernel initializer = ‘he_normal’	512
Batch Normalization		512
Activation	ReLU	512
Dropout	dropout rate = 0.5	512
…		
**Classification Layer 6**		
Fully Connected	units = 512 kernel initializer = ‘he_normal’	512
Batch Normalization		512
Activation	ReLU	512
Dropout	dropout rate = 0.5	512
Fully Connected	Softmax	2

**Table 6:** The main differences among pretrained 2D Deep CNN models that were used in this study.

Characteristic Comparison	2D Deep CNN Architecture		
	AlexNet	GoogLeNet	ResNet-50
Number of Layers	8	22	50
Input Size	227×227×3	224×224×3	224×224×3
Number of Conv-Pool Layers	5	21	49
Number of Fully Connected Layers	3	1	1
Salient Feature	Deeper	Wider (parallel kernels)	Shortcut connections
Number of Parameters (Weights)	60.97 million	7 million	25.56 million
Memory	232.5 MB	26.3 MB	97.2 MB
FLOPs	$0.7\times 10^{9}$	$1.5\times 10^{9}$	$4.1\times 10^{9}$
Training Time (5fold-CV/LOSO-CV)	2.88/20.55 h	4.56/25.41 h	4.78/28.94 h

**Table 7:** Mixed group SA per segment classification performance results using 5fold-CV.

Input System		Evaluation Parameters			
Dataset	IMF Feature	Acc (%)	Sens (%)	Spec (%)	AUC
**1D Deep CNN**					
Imbalanced	${\mathrm{IMF}}_{1}$	89.8	83.5	94	0.9629
	${\mathrm{IMF}}_{2}$	88.6	87	89.7	0.9516
	${\mathrm{IMF}}_{12}$	90.5	84.7	94.3	0.9656
	${\mathrm{IMF}}_{123}$	91 ^#^	86.1 ^#^	94.2^#^	0.9671 ^#^
Balanced using SMOTE	${\mathrm{IMF}}_{1}$	91.7	91.2	92.3	0.9721
	${\mathrm{IMF}}_{2}$	89.9	88.6	91.3	0.9622
	${\mathrm{IMF}}_{12}$	91.5	93.4	90.5	0.9714
	${\mathrm{IMF}}_{123}$	91.8 ^#^	91 ^#^	92.6^#^	0.9732 ^#^
**2D Deep CNN: AlexNet**					
Imbalanced	${\mathrm{IMF}}_{1}$	90.8	89.4	91.7	0.9665
	${\mathrm{IMF}}_{2}$	84.5	82.3	85.9	0.9182
	${\mathrm{IMF}}_{12}$	91.1 ^#^	92.2 ^#^	90.3 ^#^	0.9689 ^#^
	${\mathrm{IMF}}_{123}$	91.3	89.1	92.8	0.9684
Balanced using SMOTE	${\mathrm{IMF}}_{1}$	91.8	92	91.6	0.9730
	${\mathrm{IMF}}_{2}$	86.7	83.6	89.9	0.9399
	${\mathrm{IMF}}_{12}$	92.3 ^#^	92 ^#^	92.6 ^#^	0.9771 ^#^
	${\mathrm{IMF}}_{123}$	92	90.2	93.8	0.9779
**2D Deep CNN: GoogLeNet**					
Imbalanced	${\mathrm{IMF}}_{1}$	90.9	91	90.8	0.9678
	${\mathrm{IMF}}_{2}$	86.3	83.3	88.2	0.9344
	${\mathrm{IMF}}_{12}$	91.6 ^#^	90.1 ^#^	92.6 ^#^	0.9706^#^
	${\mathrm{IMF}}_{123}$	91.3	89	92.8	0.9684
Balanced using SMOTE	${\mathrm{IMF}}_{1}$	92.3	91.6	93.1	0.9754
	${\mathrm{IMF}}_{2}$	88.4	84.4	92.3	0.9539
	${\mathrm{IMF}}_{12}$	92.2	91	93.5	0.9780
	${\mathrm{IMF}}_{123}$	92.9 ^#^	91.9 ^#^	93.9 ^#^	0.9797 ^#^
**2D Deep CNN: ResNet-50**					
Imbalanced	${\mathrm{IMF}}_{1}$	92.2	91.3	92.7	0.9740
	${\mathrm{IMF}}_{2}$	88.8	87.8	89.4	0.9535
	${\mathrm{IMF}}_{12}$	92.5 ^#^	91.4 ^#^	93.1 ^#^	0.9758 ^#^
	${\mathrm{IMF}}_{123}$	92.3	91	93.3	0.9751
Balanced using SMOTE	${\mathrm{IMF}}_{1}$	93.4	94.3	92.5	0.9811
	${\mathrm{IMF}}_{2}$	90	92.6	87.5	0.9645
	${\mathrm{IMF}}_{12}$	93.6	94.7	92.5	0.9825
	${\mathrm{IMF}}_{123}$	93.8 ^#^	94.9 ^#^	92.7 ^#^	0.9833 ^#^

Note: ^#^ denotes the best classification result and model which selected by Youden’s index criteria.

**Table 8:** Mixed group SA per subject classification based on 1D deep CNN performance results using LOSO-CV.

**Evaluation** **Parameter**	**Subject ID**												
	**a01**	**a02**	**a03**	**a04**	**a05**	**a06**	**a07**	**a08**	**a09**	**a10**	**a11**	**a12**	**a13**
Accuracy	96	28.7	59.2	48.5	65	59.4	66.9	80.2	79.4	59.4	56.2	74.6	83.9
Sensitivity	15.8	94.9	48.1	87.2	36.9	83.5	16.1	89.1	56.1	64.5	92.9	29.3	78.7
Specificity	99.3	11.5	72.1	45.1	85.2	20.2	99	66.3	86.6	34.9	8.2	78.8	89.4
**Evaluation Parameter**	**Subject ID**												
	**a14**	**a15**	**a16**	**a17**	**a18**	**a19**	**a20**	**b01**	**b02**	**b03**	**b04**	**c01**	**c02**
Accuracy	79.6	66.9	68.5	71.1	89.8	77.1	48.3	71.7	74.2	74	95.3	6.4	49.6
Sensitivity	1	12.9	93.1	62.5	49	67.1	35.1	72.5	75.2	83.9	98	100	100
Specificity	99.5	85.7	56.2	84.1	94.6	93	56.6	52.6	69.9	20	0	6.4	49.6
**Evaluation Parameter**	**Subject ID**							**Average**					
	**c03**	**c04**	**c06**	**c07**	**c08**	**c09**	**c10**						
Accuracy	69.8	63.8	41.9	51.9	80	90.8	98.6	67.5					
Sensitivity	100	100	41.8	51.7	100	100	98.9	67.8					
Specificity	69.8	63.8	100	75	80	90.8	0	62					

**Table 9:** Mixed group SA per subject classification based on 2D deep CNN AlexNet performance results using LOSO-CV (best performance from ${\mathrm{IMF}}_{123}$).

**Evaluation** **Parameter**	**Subject ID**												
	**a01**	**a02**	**a03**	**a04**	**a05**	**a06**	**a07**	**a08**	**a09**	**a10**	**a11**	**a12**	**a13**
Accuracy	95.8	61.5	70.4	71.5	79.5	70.6	71.7	76.9	62.9	83.3	74.8	87.1	85
Sensitivity	96.7	54.9	43.7	69.8	75.8	30.6	99.7	60.4	54.9	56.6	46.7	92	79.1
Specificity	73.7	86.9	93.4	89.7	84.7	95.3	27.4	87.4	88.6	88.9	96.2	34.1	90.6
**Evaluation Parameter**	**Subject ID**												
	**a14**	**a15**	**a16**	**a17**	**a18**	**a19**	**a20**	**b01**	**b02**	**b03**	**b04**	**c01**	**c02**
Accuracy	86.9	81.9	83.5	63.3	87.9	91.9	60.2	95	92.7	92.1	95.8	98.6	100
Sensitivity	91.6	89.3	80	8	93.2	84.9	38.3	21.1	78.5	90.8	10	100	100
Specificity	68	60.5	90.6	100	43.1	96.3	95.1	98	96.1	92.4	98.3	98.6	100
**Evaluation Parameter**	**Subject ID**							**Average**					
	**c03**	**c04**	**c06**	**c07**	**c08**	**c09**	**c10**						
Accuracy	95	60.2	97.6	93.3	91.9	98.9	98.6	83.2					
Sensitivity	100	100	0	0	100	100	0	72.7					
Specificity	95	60.2	97.9	94.2	91.9	98.8	98.9	90.4					

**Table 10:** Mixed group SA per subject classification based on 2D deep CNN GoogLeNet performance results using LOSO-CV (best performance from ${\mathrm{IMF}}_{1}$).

**Evaluation** **Parameter**	**Subject ID**												
	**a01**	**a02**	**a03**	**a04**	**a05**	**a06**	**a07**	**a08**	**a09**	**a10**	**a11**	**a12**	**a13**
Accuracy	96.2	76.2	87.7	97.7	77.9	75.2	73.5	81.2	77.7	71.7	70.7	57.5	82.3
Sensitivity	78.9	76.9	79.3	98.6	81.6	44.3	95.2	82.9	88.5	86.7	34.1	55.1	71.5
Specificity	97	73.7	95	87.2	72.7	94.3	39.2	80.2	43	68.5	98.7	82.9	92.6
**Evaluation Parameter**	**Subject ID**												
	**a14**	**a15**	**a16**	**a17**	**a18**	**a19**	**a20**	**b01**	**b02**	**b03**	**b04**	**c01**	**c02**
Accuracy	89.2	84.2	81.2	62.7	85	89	78.5	96.2	90.2	82.6	87.8	95.5	98.6
Sensitivity	97.4	92.4	75.6	7.2	88.8	74.1	74.6	10.5	66.7	76.9	30	100	100
Specificity	56.7	60.5	92.5	99.5	52.9	98.3	84.9	99.8	95.9	83.7	89.4	95.5	98.6
**Evaluation Parameter**	**Subject ID**							**Average**					
	**c03**	**c04**	**c06**	**c07**	**c08**	**c09**	**c10**						
Accuracy	74.5	26.7	95.2	96	98.3	98.3	99.4	82.9					
Sensitivity	100	100	0	0	100	100	0	78.8					
Specificity	74.5	26.7	95.5	96.9	98.3	98.3	99.7	85.6					

**Table 11:** Mixed group SA per subject classification based on 2D deep CNN ResNet-50 performance results using LOSO-CV (best performance from ${\mathrm{IMF}}_{12}$).

**Evaluation** **Parameter**	**Subject ID**												
	**a01**	**a02**	**a03**	**a04**	**a05**	**a06**	**a07**	**a08**	**a09**	**a10**	**a11**	**a12**	**a13**
Accuracy	96.2	69	84	93.8	80	77.1	77.5	81.9	68.1	82.7	58.1	82.3	83.3
Sensitivity	97.8	63.5	75.2	95	75.8	50.3	95.2	74.9	60.9	66.3	4.9	84.7	77.9
Specificity	57.9	89.9	91.5	79.5	85.8	93.6	49.5	86.3	91.2	86.1	98.7	56.1	88.5
**Evaluation Parameter**	**Subject ID**												
	**a14**	**a15**	**a16**	**a17**	**a18**	**a19**	**a20**	**b01**	**b02**	**b03**	**b04**	**c01**	**c02**
Accuracy	87.9	81.9	82.1	70.5	91.7	79	61.7	94.2	89.4	76.2	95.6	96	99.5
Sensitivity	93.5	95.8	78.1	29	98.1	47	40.3	0	78.5	87.7	10	100	100
Specificity	66	41.9	90	98.1	37.3	99	95.7	98	92	74.1	98	96	99.5
**Evaluation Parameter**	**Subject ID**							**Average**					
	**c03**	**c04**	**c06**	**c07**	**c08**	**c09**	**c10**						
Accuracy	70	86.2	97.1	85.7	94.2	99.4	86.9	83.5					
Sensitivity	100	100	0	0	100	100	100	75.9					
Specificity	70	86.2	97.4	86.5	94.2	99.4	86.9	88.7					

**Table 12:** The comparison of SA classification performance with existing literature.

Framework	Author (Year)	Input System	Method	Evaluation Parameters (%)			
				Acc	Sens	Spec	AUC
DL	The proposed method	ECG	EMD + SMOTE + 1D deep CNN	91.8	91	92.6	0.9732
	Qin et al. (2022) [21]	RRI	ADASYN + 1D deep CNN + BiGRU	91.1	88.9	92.4	0.9520
	Yeh et al. (2022) [22]	ECG	Filter Bank Decomposition + 1D deep CNN	88.6	83.8	91.5	-
	Feng et al. (2021) [23]	RRI	Stacked SAE + TDCS ^1^	85.1	86.2	84.4	-
	Sheta et al. (2021) [24]	ECG	CNNLSTM	86.3	88.8	-	0.9510
	Chang et al. (2020) [25]	ECG	1D deep CNN	87.9	92	81.1	0.9400
	Singh et al. (2019) [30]	ECG	CWT + AlexNet CNN + SVM	86.2	90	83.8	0.8800
	Li et al. (2018) [53]	RRI	Stacked SAE + Decision Fusion ^2^	84.7	88.9	82.1	0.8690
ML	Lin et al. (2022) [20]	ECG	CWT + EL	91.4	89.8	92.4	-
	Bozkurt et al. (2020) [27]	ECG and HRV	Feature Engineering + Fisher + EL	85.1	85	86	-
	Surrel et al. (2018) [34]	RRI and RSA	Apnea Scoring (Energy) + SVM	85.7	81.4	88.4	-
	Hassan et al. (2017) [35]	ECG	TQWT + RUSBoost	88.9	87.6	91.5	-

Note: ^1^ TDCS consisted of MetaCost model and Softmax-HMM. ^2^ Decision fusion was the combination of parameter estimation SVM and ANN with HMM. DL: deep learning; ML: machine learning; RRI: R-R interval; HRV: heart rate variability; RSA: R-S amplitude; EMD: empirical mode decomposition; SMOTE: synthetic minority oversampling technique; ADASYN: adaptive synthetic; BiGRU: bidirectional gated recurrent unit; SAE: sparse autoencoder; TDCS: time-dependent cost-sensitive; LSTM: long-short term memory; CWT: continuous wavelet transform; SVM: support vector machine; ANN: artificial neural network; HMM: Hidden Markov Model; EL: ensemble learning; TQWT: tunable-Q factor wavelet transform; RUSBoost: random under sampling boosting; Acc: accuracy; Sens: sensitivity, Spec: specificity; AUC: area under the ROC curve.

**Table 13:** Literature survey of sleep apnea detection with data augmentation approach for dealing with imbalanced dataset problem.

Author (Year)	Input	Method		
		Feature Extraction	Data Augmentation	Classifier
Qin et al. [21] (2022)	ECG	Christov algorithm	ADASYN	1D-CNN-RLM, BiGRU-TDM
Shen et al. [54] (2021)	RRI	MSDA-1D-CNN	WCLF	WLTD (HMM)
Azimi et al. [55] (2020)	PSM	N/A	SMOTE	Linear SVM, BiLSTM, TCN
Van Steenkiste et al. [56] (2019)	Respiration	N/A	Balanced Bootstrapping	LSTM NNs + aggregated prediction
Hassan et al. [35] (2017)	ECG	TQWT + statistical features	RUSBoost	ELM, PNN, Bagging, kNN, SVM, LS-SVM, RF, AdaBoost
Hassan [57] (2016)	ECG	NIG TQWT	AdaBoost	DT

Note: N/A: not available; ADASYN: adaptive synthetic; 1D-CNN-RLM: 1D-CNN-based representation learning model; BiGRU-TDM: bidirectional gated recurrent unit-based temporal dependence model; PSM: pressure sensitive mats; MSDA-1D-CNN: multiscale dilation attention 1DCNN; WCLF: weighted cross-entropy loss function; WLTD: weighted-loss time-dependent; HMM: hidden Markov model; SMOTE: synthetic minority oversampling technique; BiLSTM: bidirectional long short-term memory; TCN: temporal convolutional network; LSTM NNs: LSTM neural networks; TQWT: tunable-Q factor wavelet transform; ELM: extreme learning machine; PNN: Prazen’s probabilistic neural network; Bagging: booststrap aggregating; kNN: k-Nearest Neighbor; SVM: support vector machine; LS-SVM: least-square SVM; RF: random forest; AdaBoost: adaptive boosting; NIG: normal inverse Gaussian; AdaBoost: adaptive boosting; DT: decision tree.

**Table 14:** The comparison SA classification performance among several data augmentation methods (based on the best SA classification model: 2D Deep CNN ResNet-50).

Balancing Method	IMF Feature	Evaluation Parameters			
		Acc (%)	Sens (%)	Spec (%)	AUC
ROS	${\mathrm{IMF}}_{1}$	93.7	95.9	91.5	0.9816
	${\mathrm{IMF}}_{2}$	90.9	93.8	88	0.9666
	${\mathrm{IMF}}_{12}$	94.1 ^#^	95.4 ^#^	92.8 ^#^	0.9834 ^#^
	${\mathrm{IMF}}_{123}$	93.8	95	92.5	0.9817
RUS	${\mathrm{IMF}}_{1}$	90.1	89.7	90.4	0.9615
	${\mathrm{IMF}}_{2}$	85.7	84.6	86.9	0.9312
	${\mathrm{IMF}}_{12}$	90.1	89.5	90.6	0.9613
	${\mathrm{IMF}}_{123}$	90.4	90.6	90.2	0.9637
SMOTE	${\mathrm{IMF}}_{1}$	93.4	94.3	92.5	0.9811
	${\mathrm{IMF}}_{2}$	90	92.6	87.5	0.9645
	${\mathrm{IMF}}_{12}$	93.6	94.7	92.5	0.9825
	${\mathrm{IMF}}_{123}$	93.8 ^#^	94.9 ^#^	92.7 ^#^	0.9833 ^#^

Note: ^#^ denotes the best classification result and model which selected by Youden’s index criteria; ROS: random over sampling; RUS: random under sampling; SMOTE: synthetic minority over sampling technique; Acc: accuracy; Sens: sensitivity; Spec: specificity; AUC: area under the ROC curve.

## Data Availability

Not applicable.
